# Imaging tests in cutaneous malignant melanoma staging: a retrospective cohort^☆^^☆☆^

**DOI:** 10.1016/j.abd.2018.12.002

**Published:** 2019-10-26

**Authors:** Luiza Boava Souza, Gabriel Peres, Juliano Vilaverde Schmitt

**Affiliations:** a Faculdade de Medicina de Botucatu, Universidade Estadual Paulista, Botucatu, SP, Brazil; b Department of Dermatology and Radiotherapy, Hospital das Clínicas, Faculdade de Medicina de Botucatu, Universidade Estadual Paulista, Botucatu, SP, Brazil

*Dear Editor*,

There is no consensus regarding the staging process of melanoma, with a diversity of protocols across the world and between different institutions in the same country.^1^

Considering the continuous increase in the incidence of melanoma and the financial demands of health systems, it is required that the management of these patients promotes good clinical results and cost–benefit ratio.^2^, ^3^

The present study evaluated the frequency of imaging tests in the staging of cutaneous melanoma patients, the rates of true and false positivity, the impact of the examination on the prognosis of the patient, and the associated demographic and clinical characteristics.

This was a retrospective cohort study analyzing medical records of cases of cutaneous melanoma treated and followed at the institution between 1999 and 2016, excluding tumors *in situ*.

Medical records in which the anatomopathological examination did not allow adequate staging of the initial tumor were excluded. Staging exams were those performed within three months of the initial diagnosis.

The results of chest radiographs (CR) and axial computed tomography (CT) of the head, neck, chest, abdomen, and pelvis were evaluated according to the radiological reports. Positivity was true when histological evidence of the neoplasm was obtained, or when the patient's evolution showed clinically evident recurrence.

Continuous variables were analyzed by Student's *t*-test and the Mann–Whitney test, depending on the normality of the distributions as assessed by the Shapiro–Wilk test. Categorical variables were compared using the chi-squared test.

The impact of radiological examination on survival from the initial diagnosis was assessed by Cox regression. Two-tailed values of *p* < 0.05 were considered significant.

We identified 93 cases included in the study, of which 57 had initial imaging tests. Table 1 shows the baseline characteristics of the patients and the imaging tests.

**Table 1:** Features of patients included in the study (*n* = 93).

Baseline features	*n* (%)
*Sex*	
Female	44 (47.3)
Male	49 (52.7)

*Age at diagnosis (years)*	62 (49–75)^a^
*Follow-up time (months)*	55 (22–88)^a^
*Death in the follow-up*	30 (32.3)
*Death by melanoma*	16 (17.2)

*Clinical type*	
Superficial spreading	44 (47.3)
Nodular	15 (16.1)
Acral lentiginous	16 (17.2)
Lentigo maligna	18 (19.4)

*Location*	
Head and neck	26 (28)
Trunk	31 (33.3)
Members	36 (38.7)
Presence of ulceration	27 (29)
Breslow thickness (mm)	1.3 (0.6–4)^a^

*Imaging tests for staging*	
Altered exams/exams performed	13/57 (22.8)
False positives findings/exams performed	9/57 (15.8)
True positive findings on chest X-rays	0/51 (0)
False positive findings on chest X-rays	3/51 (5.9)
True positive findings on CTs	3/11 (27.3)
Preclinical	2/11 (18.2)
False positive findings on CTs	6/11 (54.5)

a Median (p25–p75). CT, computed tomography.

We had a balanced sample between the sexes, with lesions treated between the sixth and eighth decade of life, followed-up for a median period of 4.6 years, and initial tumors of intermediate thickness, with 32% fatality during follow-up.

There were frequent incidental findings on initial imaging exams, with no truly positive findings at baseline CR. However, the imaging tests were performed selectively, with a greater regularity for the CRs.

Regarding the initial staging exams, only four of 57 patients presented findings indicating systemic metastasis of melanoma (through CT). All lesions were ulcerated, with a median Breslow thickness of 9.8 (1.5–15) mm, one quarter of which had clinical regional lymph node involvement, one quarter had bone invasion by the primary tumor, and one quarter had microsatellitosis.

Patients who did or did not perform initial CR did not differ in demographic and tumor staging characteristics (Table 2). In addition, general and specific mortality adjusted for age, sex, Breslow thickness, and ulceration were not different between these groups (*p* = 0.63 and *p* = 0.81 – Cox regression).

**Table 2:** Patients comparison according to the performance of chest X-rays.

Variable	Performed (*n* = 51)	Unperformed (*n* = 42)	*p*
*Sex*			0.72
Female	25 (49%)	19 (45.2%)	
Male	26 (51%)	23 (54.8%)	
*Age at diagnosis*^a^	57.4 (17.9)	63.4 (15.6)	0.09
*Breslow thickness (mm)*^b^	1.4 (0.6–4)	1 (0.5–3.5)	0.46
*Ulceration present*	14 (27.5%)	13 (31%)	0.71

a Mean (standard deviation).

b Median (p25–p75).

Of the patients with negative baseline tests, 24.5% (13/53) evolved to metastatic disease identified at follow-up imaging tests, reaching 61.5% (8/13) after clinical manifestation of relapse. Five of the 13 cases of recurrence (38.5%) presented metastatic disease in the follow-up CRs.

Of the five patients who had findings of asymptomatic metastasis on imaging examinations, three had previously progressed to stage III. The median follow-up time for pre-clinical identification of distant metastatic disease at imaging examinations was 24 (9–29) months.

Fig. 1 shows the patients’ overall survival curves from the initial diagnosis, according to the performance and alterations of the follow-up imaging tests, adjusted for age, sex, Breslow thickness, and ulceration, where more aggressive behavior of tumors with clinically identified metastatic disease is observed (*p* < 0.01 – Cox regression).

**Figure 1:**
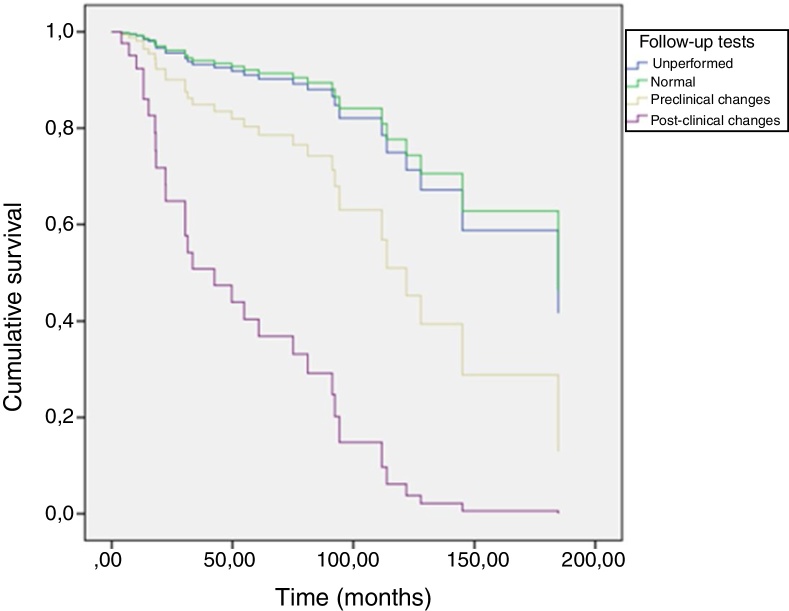
Global survival according to the performance and changes of the imaging exams during follow-up. *Including chest X-rays and axial tomography. The exams were performed selectively.

The results of the present study suggest that imaging tests to stage asymptomatic patients are not very productive and should be considered only for locally advanced cases with thick and ulcerated tumors. The studies by Gjørup et al., in 2016, and Ferrándiz et al., also in 2016, together identified only three positive cases between CR and CT in the staging of 913 asymptomatic patients up to stage IIC.^4^, ^5^

At follow-up, the frequency of positive findings in asymptomatic patients increased, but was often preceded by regional spread of the tumor. In any case, clinically manifest distant metastasis indicated a disease of aggressive behavior.

Despite the limitations inherent in retrospective studies, the results disfavored CR in the staging of asymptomatic patients, except for locally very advanced cases. During the follow-up, the positivity increases, but the tumors are usually preceded by locoregional recurrence and distant metastatic disease symptoms, within the three first years of follow-up.

## Financial support

None declared.

## Author's contribution

Luiza Boava Souza: Approval of the final version of the manuscript; conception and planning of the study; elaboration and writing of the manuscript; obtaining, analyzing and interpreting the data; critical review of the literature; critical review of the manuscript.

Gabriel Peres: Approval of the final version of the manuscript; elaboration and writing of the manuscript; critical review of the manuscript.

Juliano Vilaverde Schmitt: Statistical analysis; approval of the final version of the manuscript; conception and planning of the study; elaboration and writing of the manuscript; obtaining, analyzing and interpreting the data; effective participation in research orientation; critical review of the literature; critical review of the manuscript.

## Conflicts of interest

None declared.
